# Higher ultra processed foods intake is associated with low muscle mass in young to middle-aged adults: a cross-sectional NHANES study

**DOI:** 10.3389/fnut.2024.1280665

**Published:** 2024-02-19

**Authors:** Weiliang Kong, Yilian Xie, Jingjing Hu, Weiping Ding, Chao Cao

**Affiliations:** ^1^Key Laboratory of Respiratory Disease of Ningbo, Department of Respiratory and Critical Care Medicine, The First Affiliated Hospital of Ningbo University, Ningbo, China; ^2^Department of Hepatology, The First Affiliated Hospital of Ningbo University, Ningbo, Zhejiang, China

**Keywords:** UPFs, low muscle mass, sarcopenia, ALM, NHANES

## Abstract

**Design:**

Ultra-processed foods (UPFs) have become a pressing global health concern, prompting investigations into their potential association with low muscle mass in adults.

**Methods:**

This cross-sectional study analyzed data from 10,255 adults aged 20−59 years who participated in the National Health and Nutritional Examination Survey (NHANES) during cycles spanning from 2011 to 2018. The primary outcome, low muscle mass, was assessed using the Foundation for the National Institutes of Health (FNIH) definition, employing restricted cubic splines and weighted multivariate regression for analysis. Sensitivity analysis incorporated three other prevalent definitions to explore optimal cut points for muscle quality in the context of sarcopenia.

**Results:**

The weighted prevalence of low muscle mass was 7.65%. Comparing the percentage of UPFs calories intake between individuals with normal and low muscle mass, the values were found to be similar (55.70 vs. 54.62%). Significantly linear associations were observed between UPFs consumption and low muscle mass (*P* for non-linear = 0.7915, *P* for total = 0.0117). Upon full adjustment for potential confounding factors, participants with the highest UPFs intake exhibited a 60% increased risk of low muscle mass (OR = 1.60, 95% CI: 1.13 to 2.26, *P* for trend = 0.003) and a decrease in ALM/BMI (β = −0.0176, 95% CI: −0.0274 to −0.0077, *P* for trend = 0.003). Sensitivity analysis confirmed the consistency of these associations, except for the International Working Group on Sarcopenia (IWGS) definition, where the observed association between the highest quartiles of UPFs (%Kcal) and low muscle mass did not attain statistical significance (OR = 1.35, 95% CI: 0.97 to 1.87, *P* for trend = 0.082).

**Conclusion:**

Our study underscores a significant linear association between higher UPFs consumption and an elevated risk of low muscle mass in adults. These findings emphasize the potential adverse impact of UPFs on muscle health and emphasize the need to address UPFs consumption as a modifiable risk factor in the context of sarcopenia.

## Introduction

Ultra-processed foods (UPFs) are highly processed and industrially manufactured products that typically contain high levels of additives, sugar, salt, and unhealthy fats. These foods often undergo multiple stages of cooking, refining, and packaging to extend shelf life and provide convenience (1). They are easy to obtain and are known for their appealing taste, leading to their increasingly widespread availability and increased consumption of UPFs by more than half over the past several years (2, 3). UPFs typically consist of processed sugars, proteins, fats, as well as low-cost industrial raw materials, additives and processing methods that are rarely used in traditional cooking. They are considered to decrease the overall quality of the diet (4) and result in various health problems such as obesity (5), type-2 diabetes (6), and cardiovascular diseases (7). For this reason, public health organizations have widely expressed the need to limit UPFs consumption (8).

Sarcopenia is a medical condition closely linked to the process of aging. As individuals grow older, their metabolic functions gradually decline, leading to an inevitable reduction in muscle mass and muscle strength (9). Research indicates that without resistance training, muscle mass decline may commence after the age of 30, and by the age of 60, muscle atrophy can reach 20−40% (10). Currently, the precise definition of sarcopenia remains a topic of debate and varies across different criteria (9, 11–13). However, a prevailing characteristic among most definitions is the presence of low muscle mass (9). This particular attribute is currently recognized as a significant determinant of various health outcomes and an elevated risk of mortality (14, 15). Numerous studies have established that low muscle mass is influenced by a multitude of individual factors, encompassing genetic predisposition, birth weight, breastfeeding history, levels of physical activity, dietary habits, socio-economic statuses, and diseases (16, 17). Nowadays, resistance training is considered one of the best methods for preventing/treating sarcopenia (18). Regularly engaging in resistance training programs throughout adulthood, especially during young adulthood and midlife, can mitigate age-related changes in the musculoskeletal system and lessen their impact on metabolism and the aging process (18, 19). While dietary choices represent another crucial modifiable aspect, it is noteworthy that low muscle mass also holds significance in the diagnosis of malnutrition and constitutes one of the three phenotypic criteria considered under the auspices of the Global Leadership Initiative on Malnutrition (GLIM) criteria (20, 21). Given the marked prevalence of UPFs consumption in Western countries (2), the potential connection between UPFs intake and muscle mass has been an area of interest. Excessive consumption of UPFs not only leads to concerns about muscle health due to nutritional deficiencies and excessive intake of food additives but also evidence suggests that it may be linked to muscle-skeletal damage through the disruption of the gut microbiota ecosystem, potentially via the gut-muscle and gut-brain axes (22). While older adults are at a higher risk of sarcopenia, maintaining optimal muscle health during younger and middle-aged years is pivotal for muscle conditions in later life. Studying the relationship between muscle mass decline during these age groups and modifiable dietary patterns provides crucial insights for preventive strategies. Therefore, we conducted an assessment of the association between the proportion of UPFs in the total daily caloric intake and low muscle mass, utilizing data sourced from a nationally representative sample of U.S. adults, with measurements based on the Dual-Energy X-ray Absorptiometry (DXA) method. Our study aims to shed light on the intricate interactions between UPFs consumption and muscle health, using robust data and accounting for crucial dietary and physiological factors.

## Methods

### Design

**FIGURE 1:**
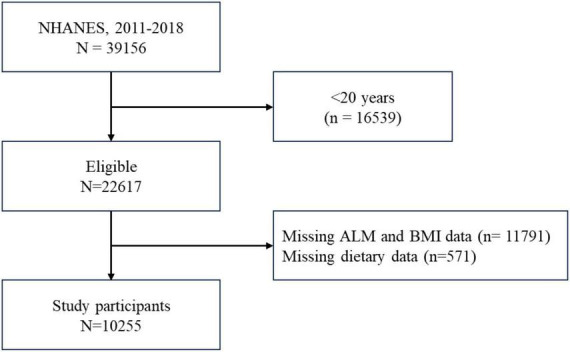
Participants flow chart.

This cross-sectional study drew upon data publicly available from the National Health and Nutrition Examination Survey (NHANES) to analyze the relationship between UPFs and muscle mass. As adults aged ≥60 years were not eligible for DXA examination, participants aged 20−59 years who responded to relevant questions concerning demographics, socioeconomic factors, dietary intake, chronic diseases, body measurements from the 2011−2018 cycles were included in the analysis. After applying additional inclusion and exclusion criteria, this left 10,255 participants available for study. Detailed information is represented in Figure 1.

### UPFs

In this study, we used the United States Department of Agriculture (USDA) Automated Multiple-Pass Method to collect 24-hour dietary recalls in-person from trained interviewers. The NOVA food classification system was applied to the Food and Nutrient Database for Dietary Studies (FNDDS) data, and all food items that matched the features of UPFs were considered. The proportion of the UPFs energy intake in total daily energy intake (%Kcal UPFs) was calculated based on the two-day average as an indicator for UPFs intake, and the UPFs consumption was divided into quartiles as the exposure variable. A more detailed description of NOVA classification can be found in Supplementary Table 1.

Previous studies have demonstrated the validity of the 24-hour dietary recall and that the UPFs classification and calorie estimation used in this study are consistent and accurate (23, 24).

Also, because many food additives cannot be measured in terms of energy, the proportion of the UPFs grams intake in total daily grams intake (%Gram UPFs) was also collected and used as an indicator for sensitivity analysis.

### Low muscle mass

Height (m), weight (kg), and body mass index (BMI) (kg/m^2^) were measured for each survey cycle conducted between 2011−2018 in the Mobile Examination Center.

All eligible participants had their appendicular lean mass (ALM) assessed using DXA. ALM, an accepted proxy for skeletal muscle mass, was calculated by summing the lean mass (excluding bone mineral content) of the right and left leg and right and left arm as measured by DXA.

Because low muscle mass is another important basis for the diagnosis of sarcopenia in addition to reduced grip strength, different definitions exist and continue to be a source of controversy. We chose the previously published definition from Foundation for the National Institutes of Health (FNIH) - low muscle mass [ALM (kg)/BMI (kg/m^2^) < 0.789 for male and <0.512 for female] as the main outcome. Besides, the updated European Working Group on Sarcopenia in Older People (EWGSOP2) (12) definition [ALM (kg)/height^2^(m^2^) < 7.0 kg/m^2^ for male and <5.5°kg/m^2^ for female], Asian Working Group on Sarcopenia (2019, AWGS2) (11) definition [ALM (kg)/height^2^(m^2^) < 7.0 kg/m^2^ for male and <5.4°kg/m^2^ for female], and International Working Group on Sarcopenia (IWGS) definition [ALM (kg)/height^2^(m^2^) < 7.23 kg/m^2^ for male and <5.67°kg/m^2^ for female] were also included for the sensitivity analysis.

### Covariates

The selection of potential confounding variables was based on prior literature findings, and these variables were subsequently adjusted in the multivariate models. These covariates included various socio-demographic characteristics, such as sex, age, race (e.g., non-Hispanic white, non-Hispanic black, Mexican American, and others), poverty income ratio [PIR, e.g., ≤1.3 categorized as low, >1.3 to ≤3.5 categorized as middle, >3.5 categorized as high (25)], marital status (e.g., never married, married or living with partner, and widowed, divorced, or separated), home status (e.g., rented, owned or being bought, and other arrangement), drinks (e.g., non-drinkers, 1−3 drinks/day, and ≥4 drinks/day), smoke status (categorized into active, former, and never based on responses to two questions: “Smoked at least 100 cigarettes in life?,” and “Do you now smoke cigarettes?”), and physical activity [categorized into inactive: less than 150 min of moderate-intensity physical activity per week, moderate: 150−300 min of moderate-intensity, or 75−150 min of vigorous-intensity aerobic physical activity per week, active: more than 300 min of moderate-intensity physical activity per week (26)]. In addition, certain chronic diseases, such as hypertension (yes or no), diabetes mellitus (DM) (yes or no), congestive heart failure (yes or no), heart attack (yes or no), stroke (yes or no), and cancer (yes or no) were included in this study. Moreover, in our multiple regression analysis, we incorporated the Urinary Albumin to Creatinine Ratio (UACR). The UACR was calculated using the following formula: UACR (mg/g) = Urinary albumin level (mg/dL)/Urinary creatinine level (g/dL). To obtain these values, spot urine samples were utilized. Additionally, estimations of glomerular filtration rate (eGFR), total energy intake (kcal), and protein intake (g) calculated as the average of 2°days were also taken into account as relevant covariates in our analysis.

## Statistical analysis

**FIGURE 2:**
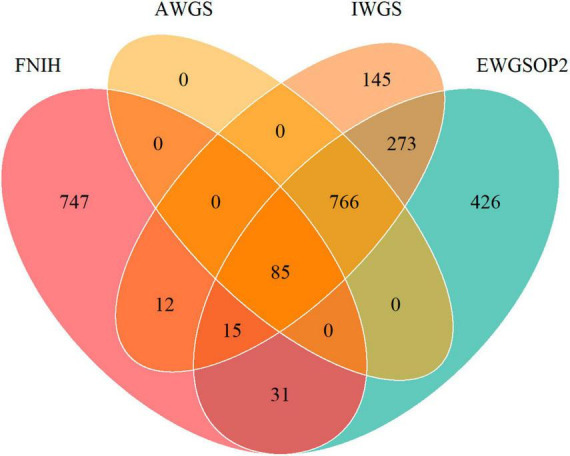
Venn diagram showing the overlap of prevalence of low muscle mass by different definitions of FNIH, EWGSOP2, AWGS, and IWGS.

The NHANES is a population-based survey that utilizes a non-random, stratified sampling design in order to effectively represent specific population subgroups. Sample weights are assigned to participants to account for non-response and other complexities associated with survey design. Our analysis follows the guidelines of NHANES, merging data from four separate cycles (2011−2018) into one 8-year dataset. In order to approximate standard errors for all continuous variables, we employed a Taylor Series Linearization approach. Subsequently, Student’s *t*-test was used to study associations of categorical variables, while weighted percentages, means [95% confidence intervals (CI)], and survey-weighted chi-squared tests were used to analyze categorical variables. Furthermore, we utilized restricted cubic splines, with four knots (20th, 40th, 60th, 80th percentiles) to flexibly model and visualize the relationship between UPFs and low muscle mass in multivariable logistic regression models. There was no evidence of departure from linearity observed in our analysis (Figure 2). Multiple regression models were then employed to calculate the adjusted odds ratio (OR) of low muscle mass across the quartile of UPFs (%Kcal) consumption. The adjusted differences of ALM/BMI across the quartile of UPFs (%Kcal) consumption were also estimated. Subgroup and interactive analyses were carried out in order to explore differences between selected subgroups.

Sensitivity analyses were then implemented in order to further explore the association between UPFs consumption and low muscle mass. The initial sensitivity analysis investigated the correlation between the proportion of UPFs by weight and muscle mass. This approach was motivated by previous studies suggesting that evaluating the percentage of UPFs based on the total weight of consumed foods and beverages might provide a more comprehensive understanding. By considering the weight of UPFs in relation to the entire diet, it allows for the inclusion of UPFs that do not contribute significantly to energy intake (e.g., artificially sweetened beverages) and those directly influenced by food processing characteristics rather than their nutritional attributes (27, 28). The intake of UPFs (%Gram) was categorized into quartiles, and its association with β value and ORs for muscle mass and low muscle mass was assessed (Supplementary Table 2). The second sensitivity analysis examined the association between the UPFs (%Kcal) and different low muscle mass definitions (AWGS2, EWGSOP2, and IWGS), as there are still debates about the cut point of low muscle mass for sarcopenia.

All data analysis was performed using software R (version 4.3.1) and the “survey” package.

## Results

Our study encompassed a total of 10,255 participants (NHANES 2011-2018 cycles). The weighted prevalence of low muscle mass (FNIH) was found to be 7.65%. When comparing the consumption of UPFs in terms of both %Kcal and %Gram between individuals with normal muscle mass and those with low muscle mass, the proportions were similar, with values of 55.70% ± 0.38% versus 54.62% ± 1.11 (*P* = 0.3608) for %Kcal and 36.11% ± 0.36% versus 38.41% ± 1.10% (*P* = 0.0607) for %Gram, respectively. Upon further examination, individuals with low muscle mass exhibited several distinguishing characteristics in comparison to those with normal muscle mass. Specifically, the former group tended to be older, male, and with lower income as indicated by low PIR. Moreover, they were more likely to have completed education up to middle school or lower and high school, and were less likely to be regular consumers of alcoholic beverages. Additionally, individuals with low muscle mass had higher prevalence rates of various health conditions, including stroke, heart attack, congestive heart failure, coronary heart disease, angina, hypertension, and diabetes mellitus (DM). Moreover, they demonstrated lower total energy intake and protein intake, as well as reduced eGFR. On the other hand, their BMI and weight were higher. For further insight, the detailed demographic and behavioral characteristics of the study participants are provided in Table 1. And Supplementary Table 3 showed the characteristics of the study participants by quartiles of UPFs (%Kcal). Figure 2 depicts a Venn diagram illustrating the prevalence of low muscle mass as determined by various definitions.

**TABLE 1:** Characteristics of the study participants by normal and low muscle mass among U.S adults aged 20–59 years.

Variable	Total	Normal	Low	*P* value
	***N* = 10255**	***N* = 9365**	***N* = 890**	
**Age**	39.27 (0.26)	38.95 (0.28)	43.09 (0.54)	<0.001
**Sex**				0.04
Female	49.79 (0.02)	50.19 (0.69)	44.98 (2.35)	
Male	50.21 (0.02)	49.81 (0.69)	55.02 (2.35)	
**Race/ethnicity**				<0.001
Non-Hispanic White	60.01 (0.03)	61.02 (1.99)	47.85 (2.78)	
Non-Hispanic Black	11.60 (0.01)	12.28 (1.05)	3.49 (0.57)	
Mexican American	10.89 (0.01)	9.72 (1.00)	24.99 (3.06)	
Others	17.50 (0.01)	16.98 (0.90)	23.68 (1.98)	
**Marital status**				0.1
Never married	26.03 (0.01)	26.23 (1.19)	23.65 (2.32)	
Married or living with partner	60.37 (0.03)	60.48 (1.19)	59.17 (2.66)	
Widowed, divorced, or separated	13.59 (0.01)	13.29 (0.63)	17.18 (1.92)	
**PIR**				<0.001
Low	23.01 (0.01)	23.71 (1.13)	35.24 (2.03)	
Middle	32.09 (0.02)	34.02 (1.09)	37.59 (2.59)	
High	38.48 (0.02)	42.26 (1.51)	27.17 (2.62)	
**Education**				<0.001
College or more	65.15 (0.03)	66.61 (1.42)	47.66 (2.62)	
Middle school or lower	3.70 (0.00)	3.05 (0.31)	11.52 (1.44)	
High school	31.14 (0.01)	30.34 (1.32)	40.82 (2.64)	
**Physical activity**				<0.001
Active	59.75 (0.02)	60.72 (0.72)	48.00 (2.45)	
Inactive	12.85 (0.01)	12.74 (0.55)	14.18 (1.77)	
Moderate	10.74 (0.01)	10.84 (0.50)	9.51 (1.46)	
Others	16.66 (0.01)	15.70 (0.60)	28.30 (2.15)	
**Home status**				0.01
Rented	37.83 (0.02)	38.01 (1.43)	43.77 (2.48)	
Owned or being bought	58.15 (0.03)	59.61 (1.51)	53.02 (2.29)	
Other arrangement	2.40 (0.00)	2.37 (0.27)	3.20 (1.25)	
**Smoke status**				0.53
Now	21.83 (0.01)	22.02 (0.79)	19.56 (1.92)	
Former	19.29 (0.01)	19.21 (0.73)	20.35 (2.25)	
Never	58.85 (0.02)	58.77 (0.93)	60.09 (2.66)	
**Drinks**				<0.001
Non-drinkers	22.95 (0.01)	21.85 (0.97)	36.31 (1.72)	
1−3 drinks/day	57.06 (0.02)	58.32 (1.15)	41.84 (2.37)	
≥4 drinks/day	19.99 (0.01)	19.83 (0.87)	21.85 (2.14)	
Cancer	5.097 (0.004)	4.96 (0.32)	6.79 (1.47)	0.17
Stroke	1.264 (0.001)	1.12 (0.13)	2.98 (0.96)	0.01
Heart attack	1.213 (0.001)	0.98 (0.13)	4.03 (0.81)	<0.001
Congestive heart failure	0.772 (0.001)	0.72 (0.10)	1.38 (0.41)	0.06
Coronary heart disease	0.951 (0.001)	0.83 (0.14)	2.47 (0.75)	0.001
**Age**	39.27 (0.26)	38.95 (0.28)	43.09 (0.54)	<0.001
Angina	0.980 (0.001)	0.87 (0.14)	2.37 (0.96)	0.03
Hypertension	27.26 (0.01)	25.98 (0.76)	42.66 (2.20)	
DM	8.86 (0.00)	7.83 (0.37)	21.29 (1.73)	<0.001
Energy (kcal)	2241.16 (11.65)	2259.87 (11.97)	2015.46 (40.35)	<0.001
Protein (g)	86.24 (0.63)	86.95 (0.65)	77.65 (1.74)	<0.001
eGFR	101.57 (0.39)	101.27 (0.39)	105.26 (0.92)	<0.001
BMI	28.71 (0.13)	28.19 (0.12)	34.88 (0.36)	<0.001
Weight (kg)	82.01 (0.36)	81.33 (0.35)	90.18 (1.14)	<0.001
UPFs (%Kcal)	55.61 (0.36)	55.70 (0.38)	54.62 (1.11)	0.3608
UPFs (%Gram)	36.29 (0.33)	36.11 (0.36)	38.41 (1.10)	0.0607

Weighted Mean ± Se and Student’s *t*-test for continuous variables. Weighted%, mean (95% CI), and Cochran-Mantel-Haenszel Chi-square test for categorical variables.

**FIGURE 3:**
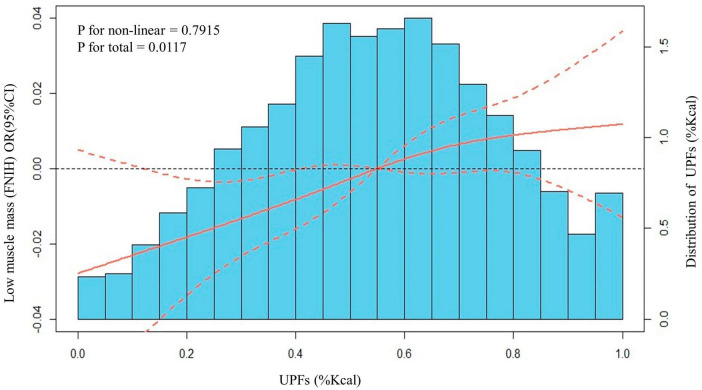
Distributions of frequency of UPFs (%Kcal) and dose–response relationship between UPFs (%Kcal) and low muscle mass in US adults 20–59 years (*n* = 10,255), NHANES 2011 to 2018. Values represent difference in predicted response in reference to a UPFs (%Kcal) of mean. Red solid lines and Red dotted line represent restricted cubic spline models and 95%CI, respectively. Multivariable logistic regression model is used to estimate the fully adjusted OR in low muscle mass (FNIH definition) and corresponding 95% CI. Model was adjusted by age, ethnicity, PIR, marital status, home status, education, physical activity, smoke status, drinks, eGFR, UACR, hypertension, DM, angina, coronary heart disease, congestive heart failure, heart attack, stroke, cancer, energy (Kcal), and protein (g).

The dose-response relationships between UPFs, expressed as a percentage of daily caloric intake, and the occurrence of low muscle mass was also estimated. To explore these relationships, we employed both linear and spline models, accounting for all potential confounding factors. Figure 3 serves as a visual representation of these dose-response relationships, capturing the interplay between UPFs and low muscle mass. Our meticulous analysis revealed a notable linear association between UPFs and low muscle mass (*P* for non-linear = 0.7915, *P* for total = 0.0117). This implies that as the proportion of UPFs in the daily caloric intake increases, there is a corresponding impact on the occurrence of low muscle mass.

Table 2 presents the weighted OR with 95%CI for low muscle mass categorized into quartiles of UPF calorie intakes, accounting for relevant covariates. Following adjustments for all selected covariates using multiple logistic regression (model 3), it was found that participants with the highest UPFs (%Kcal) intake faced a 60% higher risk of low muscle mass (OR = 1.60, 95% CI: 1.13 to 2.26, *P* for trend = 0.003) compared with the lowest quartile. Additionally, we explored the relationship between UPFs and ALM/BMI. After accounting for the specified covariates, it was observed that the highest UPFs intake compared to individuals with the lowest UPFs intake, there was a significant reduction in ALM/BMI (β = −0.0176, 95% CI: −0.0274 to −0.0077, *P* for trend = 0.03).

**TABLE 2:** Multivariable associations between quartiles of UPFs (%Kcal) and low muscle mass defined by FNIH.

Characters	Quartiles of UPFs (%Kcal)				*P* for trends
	**Q1**	**Q2**	**Q3**	**Q4**	
**ALM/BMI**					
Model 1	Ref.	0.0272 (0.0103, 0.0440)	0.0264 (0.0103, 0.0425)	0.0247 (0.0108, 0.0385)	0.004
Model 2	Ref.	0.0035 (−0.0048, 0.0117)	0.0048 (−0.0091, 0.0108)	−0.016 (−0.0263, −0.0057)	0.003
Model 3	Ref.	−0.0048 (−0.0129, 0.0032)	−0.0048 (−0.0147, 0.0052)	−0.0176 (−0.0274, −0.0077)	0.003
**Low muscle mass (FNIH)**					
Model 1	Ref.	0.79 (0.63, 1.00)	0.83 (0.61, 1.13)	0.86 (0.63, 1.16)	0.384
Model 2	Ref.	1.01 (0.80, 1.27)	1.13 (0.85, 1.52)	1.35 (0.98, 1.86)	0.047
Model 3	Ref.	1.17 (0.87, 1.57)	1.47 (1.06, 2.04)	1.60 (1.13, 2.26)	0.003

Model 1: adjusted for none.

Model 2: adjusted for age and race/ethnicity. ALM/BMI was additionally adjusted for sex.

Model 3: adjusted for age, ethnicity, PIR, marital status, home status, education, physical activity, smoke status, drinks, eGFR, UACR, hypertension, DM, angina, coronary heart disease, congestive heart failure, heart attack, stroke, cancer, energy (kcal), and protein (g). ALM/BMI was additionally adjusted for sex. Ref, reference.

Subsequently, we performed subgroup analyses and assessed potential interactions (Table 3). The results indicated that the associations between UPFs consumption and low muscle mass remained generally consistent across the selected subgroups, demonstrating stability in their patterns.

**TABLE 3:** The association between UPFs (%Kcal) and low muscle mass, stratified by subgroups.

Character	Quartiles of UPFs (%Kcal)				*P* *
	**Q1**	**Q2**	**Q3**	**Q4**	
**Age**					0.25
<40	Ref.	0.95 (0.56, 1.63)	0.95 (0.59, 1.53)	1.37 (0.82, 2.29)	
40−59	Ref.	1.22 (0.83, 1.79)	1.57 (1.05, 2.34)	1.28 (0.80, 2.03)	
**Race/ethnicity**	Ref.				0.42
Non-Hispanic White		1.31 (0.74, 2.34)	1.32 (0.72, 2.42)	1.68 (0.96, 2.95)	
Non-Hispanic Black	Ref.	0.64 (0.27, 1.52)	0.63 (0.24, 1.65)	0.35 (0.13, 0.94)	
Mexican American	Ref.	1.01 (0.62, 1.65)	1.37 (0.87, 2.16)	1.37 (0.62, 2.99)	
Others	Ref.	0.86 (0.53, 1.39)	1.45 (0.84, 2.52)	1.10 (0.59, 2.05)	
**PIR**					0.95
Low	Ref.	0.98 (0.56, 1.69)	1.41 (0.86, 2.30)	1.18 (0.61, 2.28)	
Middle	Ref.	1.13 (0.66, 1.94)	1.23 (0.67, 2.28)	1.17 (0.69, 1.98)	
High	Ref.	0.96 (0.48, 1.95)	1.34 (0.60, 2.99)	1.67 (0.81, 3.44)	
**Marital status**					0.39
Never married	Ref.	1.57 (0.63, 3.93)	2.01 (0.83, 4.85)	1.15 (0.46, 2.89)	0.12
Married/Living with partner	Ref.	1.01 (0.68, 1.50)	1.18 (0.75, 1.87)	1.64 (1.00, 2.71)	
Widowed/Divorced/Separated	Ref.	1.06 (0.42, 2.64)	1.31 (0.48, 3.59)	1.08 (0.43, 2.70)	
**Home status**					0.06
Rented or others	Ref.	1.63 (1.13, 2.33)	1.68 (1.11, 2.54)	1.34 (0.86, 2.10)	
Owned or being bought	Ref.	0.77 (0.45, 1.34)	1.07 (0.65, 1.75)	1.35 (0.79, 2.30)	
**Education**					0.33
College or more	Ref.	0.86 (0.53, 1.41)	1.12 (0.71, 1.77)	1.29 (0.76, 2.16)	
Middle school or lower	Ref.	2.33 (1.15, 4.70)	1.83 (0.75, 4.47)	1.62 (0.60, 4.37)	
High school	Ref.	1.19 (0.79, 1.79)	1.34 (0.84, 2.13)	1.19 (0.74, 1.91)	
**Physical activity**					0.13
Others	Ref.	1.33 (0.75, 2.35)	1.35 (0.74, 2.48)	1.35 (0.65, 2.79)	
Active	Ref.	0.96 (0.57, 1.64)	1.44 (0.89, 2.34)	1.01 (0.58, 1.76)	
Inactive	Ref.	1.69 (0.72, 3.97)	0.86 (0.38, 1.93)	1.57 (0.61, 4.02)	
Moderate	Ref.	0.66 (0.31, 1.41)	1.79 (0.64, 5.00)	2.89 (1.07, 7.80)	
**Smoke status**					0.19
Now	Ref.	0.79 (0.35, 1.78)	1.13 (0.53, 2.43)	0.76 (0.37, 1.57)	
Former	Ref.	1.45 (0.69, 3.05)	2.35 (1.19, 4.65)	1.86 (0.89, 3.90)	
Never	Ref.	1.11 (0.76, 1.60)	1.08 (0.71, 1.64)	1.46 (0.99, 2.15)	
**Drinks**					0.08
Non-drinkers	Ref.	0.84 (0.49, 1.44)	0.99 (0.56, 1.73)	1.19 (0.69, 2.08)	
1−3 drinks/day	Ref.	1.27 (0.85, 1.90)	1.71 (1.01, 2.88)	1.99 (1.26, 3.14)	
≥4 drinks/day	Ref.	1.38 (0.63, 3.00)	1.32 (0.67, 2.61)	0.67 (0.27, 1.65)	
**Hypertension**					0.3
No	Ref.	1.01 (0.71, 1.44)	1.06 (0.74, 1.54)	1.24 (0.85, 1.82)	
Yes	Ref.	1.29 (0.77, 2.19)	1.88 (0.98, 3.59)	1.46 (0.80, 2.67)	
**DM**					0.54
No	Ref.	1.09 (0.75, 1.58)	1.32 (0.92, 1.89)	1.24 (0.85, 1.80)	
Yes	Ref.	1.28 (0.73, 2.27)	1.27 (0.67, 2.43)	1.94 (0.88, 4.26)	

Models were adjusted by age, ethnicity, PIR, marital status, home status, education, physical activity, smoke status, drinks, eGFR, UACR, hypertension, DM, angina, coronary heart disease, congestive heart failure, heart attack, stroke, cancer, energy (kcal), and protein (g). The subgroup variable was not included in same subgroup analysis.

P*, P for interaction.

Moreover, in our sensitivity analyses, we undertook an examination of the relationship between UPFs consumption (%Gram) and low muscle mass, adhering to the definition provided by the FNIH. Additionally, we assessed the association between UPFs consumption (%Kcal) and low muscle mass using the criteria established by the AWGS2, EWGSOP2, and the IWGS. Across these sensitivity analyses, the fundamental patterns of association remained largely unchanged, except for the results obtained with the IWGS definition. In the case of IWGS, the observed association between the highest quartiles of UPFs (%Kcal) and low muscle mass did not attain statistical significance (OR = 1.35, 95% CI: 0.97 to 1.87, *P* for trend = 0.082) (Supplementary Tables 2, 4).

## Discussion

Data from the NHANES nationally representative sample of adults aged 20−59 years reveals that UPFs constitute a noteworthy component of the adult diet and exhibit a linear correlation with low muscle mass. This correlation remains consistent across various cut points defining low muscle mass of sarcopenia context except for IWGS definition. Consequently, these findings imply that increased UPF consumption negatively impacts muscle mass in adults and represents a significant driving factor for sarcopenia.

Advances in the field of aging biology have shed light on the complex underlying processes involving myocyte, inflammatory, and hormonal mechanisms, contributing to body fat redistribution, decline in lean muscle mass, and reduced muscle strength (29, 30). Traditionally, sarcopenia has been primarily assessed based on muscle mass and grip strength. However, the heterogeneity within the sarcopenia patient population has led to controversies among professional societies, resulting in variations in cutoff values for grip strength and muscle mass, impacting the design of targeted interventions (31, 32). Furthermore, since muscle mass is one of the three criteria for diagnosing malnutrition, the consumption of UPFs, known for their low dietary quality, may increase the risk of malnutrition in adults.

Ultra-processed foods (UPFs) are gradually penetrating our traditional diets and have become dominant in high- and middle-income countries (2, 33). In the younger and middle-aged population, the integration of professional and personal responsibilities, coupled with a fast-paced work-life style, may inadvertently lead to increased sedentary behavior. Additionally, the appeal of UPFs’ convenience and extensive advertising further encourages individuals to choose these highly processed food options. Numerous observational studies have indicated that high UPF intake in adults is associated with various metabolic risk factors, including heightened body weight and BMI (34), elevated total cholesterol and low-density lipoprotein levels (35), and metabolic syndrome (23, 27). In France, the NutriNet-Santé cohort (27) survey further confirmed that higher UPFs consumption correlated with increased BMI and a greater risk of overweight or obesity. While epidemiological evidence on the association between UPFs and bone composition is lacking, animal studies have demonstrated that young rats consuming UPFs with high fat and sugar content experience growth retardation, decreased bone mineral density, damage to bone structural components, and growth plate impairment (36). Considering the potential links between body composition, fat mass, bone mass, and muscle mass (29), there is plausible reason to suspect that UPFs may adversely affect muscle mass. Previous research has revealed significant associations between UPFs and various markers of muscle health in different populations. Studies conducted in China involving adults over 40 years of age demonstrated a negative correlation between UPFs and grip strength (37), while investigations in Brazilian adolescents found associations between UPFs and body composition, particularly lean body mass (38). Furthermore, studies in Brazilian adults indicated that UPFs were linked to specific markers of muscle mass, such as arm circumference (39). These findings are consistent with our prior assumptions, which have been further corroborated by the current study, indicating a negative relationship between UPFs and muscle mass in adults.

However, when we reassessed the cutoff for low muscle mass based on different criteria for sarcopenia, we observed that only 9.6% of individuals were defined as having low muscle mass in all four definitions, whereas 16.3% of individuals in the IWGS definition were not classified as having low muscle mass in the other three definitions (Figure 2). Such substantial discrepancies result in considerable classification deviations among individuals and render the conclusions inconsistent. On one hand, this suggests that the diagnosis of sarcopenia necessitates additional data updates and support. On the other hand, the application of corresponding definitions should consider regional and population differences to avoid resulting biases (40).

The relationship between UPFs and the risk factors associated with low muscle mass is underpinned by several potential mechanisms. Previous studies have shed light on the impact of various dietary components, such as dietary fiber, red meat, oily fish, retinol, magnesium, and vitamins, on muscle mass and strength (41–44). For example, an adequate intake of protein, especially leucine-rich protein, can stimulate muscle protein synthesis and serve a preventive or interventional role in individuals facing muscle loss (45). Moreover, Omega-3-rich foods like fish, nuts, and grains possess anti-inflammatory properties that impede white blood cell migration toward sources of inflammation and hinder cell aggregation, thereby contributing to the preservation of muscle mass (46). Additionally, dietary fiber has been found to mitigate oxidative stress and inflammation. Conversely, UPFs often lack these crucial nutrients and are characterized by elevated levels of sugar, sodium, trans fats, saturated fats, and inadequate protein and essential nutrients (47). Furthermore, the process of over-processing UPFs, coupled with the addition of sugars and modifications to food additives and compositions, leads to overeating and an imbalance in intestinal flora. Research by Suez et al. (48) has substantiated that non-caloric artificial sweeteners can disrupt microbial metabolic pathways. Additionally, dietary emulsifiers, such as lecithin, fatty acid monoglycerides, and diglycerides, have been shown to enhance bacterial translocation across epithelial cells *in vitro*, promoting systemic inflammation (48, 49). This phenomenon reduces the diversity of intestinal microorganisms, decreases the abundance of Bacteroides, and increases the abundance of mucin-degrading microorganisms (*Akkermansia muciniphila*) and *Proteobacteria*. Furthermore, various other types of food additives, such as preservatives, nanoparticles, foaming agents, stabilizers, flavor enhancers, and others, individually or in combination with each other or with different types of food additives, have been found to potentially confer growth advantages or increased toxicity in host gut microbiota, including (opportunistic) pathogenic micro-organisms, leading to dysbiosis (50, 51). These changes in the microbiota contribute to disrupting the intestinal barrier’s function, compromising nutrient absorption, and increasing the glycemic load, thereby affecting overall metabolic function (52, 53). Additionally, compromised permeability, also known as “gut leaky syndrome” may lead to higher endotoxemia as harmful substances enter, thereby promoting systemic inflammation and a chronic state of immune activation. Ultimately, these processes may affect skeletal muscle through the gut-muscle axis or gut-brain axis, increasing the risk of musculoskeletal injury (22). One common scenario is when athletes increase their intake of sports energetic supplements for performance and recovery, potential microbiota dysbiosis and leaky gut may lead to gastrointestinal complications, a significant factor in poor endurance performance and dropout (54). Moreover, environmental pollutants from food packaging, such as phthalates and bisphenol, may also impact muscle mass and strength (55). Unfortunately, to date, research on the relationship between dietary patterns and sarcopenia has primarily focused on the elderly (>70 years old) (56). Limited evidence exists regarding the potential impact of overall diet quality on muscle mass and function decline in younger and middle-aged individuals. Some studies suggest that dietary patterns can affect muscle mass and function in middle-aged populations through inflammation, oxidative stress, and metabolic mechanisms (57). For instance, there is an association between pro-inflammatory diets and muscle mass decline in middle-aged individuals (58), and UPFs is linked to grip strength reduction in adults over 40 years old (37). Furthermore, there are indications suggesting that a higher quality diet during middle age may be beneficial for muscle mass and function 10−20 years later (58). The precise pathogenesis of skeletal muscle mass loss due to nutrition remains incompletely understood, highlighting the need for further research, particularly prospective intervention studies involving healthy young and middle-aged populations. Studies should specifically focus on cumulative exposure to dietary patterns to provide preventive opportunities.

This study, the first of its kind, assesses the association between UPFs and low muscle mass in a large, nationally representative sample of U.S. adults aged 20−59 years. The analysis considered individual consumption data, taking into account both caloric intake ratios and weight-defined UPF intake. The use of the DXA method for muscle mass measurement in NHANES addresses previous shortcomings related to body surface markers in other studies. Furthermore, the study considered multiple definitions of sarcopenia diagnosis from different guidelines, demonstrating a high level of agreement.

This study was limited in that it was a cross-sectional study which precludes the ability to make causal inferences. Additionally, while a 5-step interview was conducted, the accuracy of the type and amount of food intake was largely dependent on the participants’ recollections. Studies have indicated that 24-hour dietary recall may lead to an underestimation of energy intake by up to 11% (59), though this likely does not affect the dietary contributions established by the study. Furthermore, UPFs were classified by NOVA and some misclassification bias may have arisen due to the fact that the NHANES dietary survey was not designed to distinguish according to the NOVA system. Lastly, the simple classification of physical activity, may have overestimated the strength of the association due to the fact that higher UPF consumption often correlates to an overall unhealthy lifestyle, including a lessened daily activity and exercise.

## Conclusion

In conclusion, this large-scale cross-sectional study establishes a significant linear correlation between UPFs and low muscle mass. The results underscore the significance of dietary pattern interventions in promoting optimal muscle health. Considering a healthier and higher-quality diet throughout the entire adult lifespan, associated with known benefits for various health outcomes, including the effective maintenance of muscle mass and function, reducing UPF intake could serve as an effective strategy to prevent low muscle mass in young and middle-aged adults, potentially contributing to better physical function in older age.

## Data availability statement

The original contributions presented in this study are included in this article/Supplementary materials, further inquiries can be directed to the corresponding authors.

## Ethics statement

The studies involving humans were approved by the CDC/NCHS Research Ethics Review Board. The studies were conducted in accordance with the local legislation and institutional requirements. The participants provided their written informed consent to participate in this study.

## Author contributions

WK: Conceptualization, Data curation, Supervision, Visualization, Writing – original draft, Writing – review & editing. YX: Methodology, Validation, Writing – original draft. JH: Data curation, Formal Analysis, Writing – review & editing. WD: Investigation, Project administration, Software, Writing – review & editing. CC: Conceptualization, Supervision, Writing – review & editing.
