# Supplementary material for: Higher ultra processed foods intake is associated with low muscle mass in young to middle-aged adults: a cross-sectional NHANES study
Source: Front Nutr. 2024 Feb 19;11:1280665. doi: 10.3389/fnut.2024.1280665 (PMC10909937; doi:10.3389/fnut.2024.1280665)
Supplement: Supplementary file 1 [file Data_Sheet_1.docx]

**Online Supplementary Material**

**Ultra-processed foods Classified in NHANES by NOVA**

NOVA classification

The NOVA classification categorizes foods and beverages into 4 groups based on the degree of industrial processing. And category 4 is ultra-processed foods (UPFs) which have undergone huge separation from nature. Detailed information on the NOVA classification is shown below. Category 1. Unprocessed or minimally processed foods are foods that have not been altered from their natural state or have only undergone processes like removal of inedible or unwanted parts, fractioning, grinding, drying, fermentation, pasteurization, roasting, boiling, cooling, or freezing. The purpose of this processing is to preserve or keep the freshness of natural foods, to make foods safe or edible, or more pleasant to consume. These foods do not contain added substances such as salt, sugar, oils, or fats, but may infrequently contain preservatives. Many unprocessed or minimally processed foods are prepared and cooked at home or in restaurant kitchens in combination with processed culinary ingredients as dishes or meals. Examples include whole grains, millets, wheat flour, fresh or frozen fruits and vegetables, unprocessed meats and poultries, eggs, fish, fresh and pasteurized milk, unflavored yogurt, legumes, nuts, and seeds. Category 2. Processed culinary ingredients are substances extracted from the first NOVA group or nature by processes such as pressing, grinding, crushing, pulverizing, and refining. These ingredients are used for seasoning and cooking foods in the first group. Examples include salt, sugar, honey, vegetable oils, butter, lard, vinegar, maple syrup (100%), molasses, and honey. Category 3. Processed foods are products manufactured by industry by adding salt, sugar, oil, or other NOVA group 2 ingredients to unprocessed or minimally processed foods to preserve or make them more palatable. These foods may contain preservatives, antioxidants, and stabilizers. Examples include canned/bottled vegetables and legumes, fruits in syrup, canned fish and meats, cheeses, salted or sugared nuts and seeds, and bread made of ingredients used in culinary preparations (i.e., wheat flour, yeast, water, salt, butter, or sugar). Category 4. UPFs are ready-to-eat/drink/heat industrial formulations that are made with multiple industrial ingredients extracted from foods or synthesized in laboratories while containing little whole foods. Besides salt, sugar, oils and fats, and preservatives, ultra-processed foods include ingredients not used in culinary preparations, in particular, flavors, colors, sweeteners, emulsifiers, and other additives used to imitate sensorial qualities of unprocessed or minimally processed foods or to disguise undesirable aspects of the final product. The processes for making ingredients or final products of ultra-processed foods may include hydrogenation and hydroxylation, extrusion and molding, and pre-processing for frying. The overall purpose of ultra-processing is to create highly profitable, hyper-palatable ready to consume products with long shelf-life. Ultra-processed food products are usually packaged attractively and marketed intensively. Examples include carbonated drinks, fruit-flavored drinks, sausages, biscuits, sweet/savory packaged snacks, candies, ready-to-eat/heat pizza, sandwiches, or burgers, frozen or shelf-stable dishes, and instant soups/noodles.

**The application of NOVA in NHANES**

The main food description and corresponding ingredient list include abbreviated descriptions (up to 60 characters) and complete descriptions (up to 200 characters) associated with each USDA food code identified in the Individual Foods files. And, each NHANES food code was obtained from the cycle-specific USDA Food and Nutrition Database for Dietary Studies (FNDDS) and the USDA National Nutrient Database for Standard Reference (SR). According to “Main food Description”, foods include bakery products such as cakes/cookies/pies, sauce, chips, chocolate, and mixed dishes such as sandwiches, burgers, pizza, and pasta dishes. These foods were classified directly as ultra-processed foods. Also, if they were indicated as branded, pre-prepared or ready-to-eat/heat/drink products were classified as ultra-processed foods. If no evidence of ultra-processing is identified for the food code, classification is applied to the basic ingredient (SR code) of the food code and discussed to achieve a more accurate classification. That means the lack of sufficient information to determine the degree of processing is usually solved by selecting a lower degree of processing. For example, the food code "Yogurt, NFS" is classified as unprocessed/minimally processed food. Classify the self-made formulas of unknown ingredients according to the expected principal components. For example, SR Code "Restaurant, Chinese, Sesame Chicken" was coded as "Orange chicken" in the past, and it was classified as "meat" among unprocessed/minimally processed foods. This may slightly underestimate the consumption of processed cooking materials or UPFs. Finally, according to the "combined food type" and "food source" of each food recorded in NHANES, the classification was reviewed and necessary modifications were made. In this step, most foods consumed as "frozen foods" or "lunches" or purchased in vending machines or fast-food restaurants are reclassified as UPFs. More detailed information is listed in supplemental Table 1.

**Supplemental Table 1. Ultra-Processed Food Subgroups and Examples**

| **Ultra-Processed Food Subgroups** | **Example Foods** |  |
| --- | --- | --- |
| **Bread and other grain foods** | Bread, rolls, biscuits, breakfast cereals: that contains classes of additives whose function is to make the final product palatable or appealing, such as flavors or colors, and is not homemade or acquired from bakery store (mainly determined by "combined food type" and "food source"). |  |
| **Ready-to-eat/heat mixed dishes** | Ready-to-eat/heat pizza, sandwiches and burgers on a bun, and Other mixed dishes: Meat/seafood/poultry/egg mixed dishes, grain-based mixed dishes (pasta dishes, rice dishes, macaroni and cheese, turnovers, and other), Mexican mixed dishes, Asia mixed dishes, and soups, when indicated as frozen, microwaved, prepared, canned, boxed, fast food, preheated, or contain non-culinary ingredients. |  |
| **Snacks and Sweets** | Snacks, candies, cereal, nutrition bars, Ice cream, savory, chocolate; ready-to-eat or dry mixed dairy desserts (such as pudding), fruit desserts; jellies and jams and preserves; toppings; gelatin desserts; fries, hash browns, potato puffs, stuffed potatoes |  |
| **Meat, poultry, and fish products** | | Fast food meat patties/fish sticks, patties, or fillets; sausages, lunchmeats; meat spreads; |
| **Condiment and Sauces** | Industrial fats, margarine, cream substitutes, cheese spread; salad dressings, tomato-based/soy-based/other condiments; dips,  gravies, and other sauces |  |
| **Beverages and Flavored foods** | All beverages except fresh fruit juice; Flavored milk, yogurts; Milkshake and other dairy drinks, dairy substitutes such as almond milk, coconut milk, rice drink, soy milk |  |
| **Others** | Sweeteners, and all syrups (excluding 100% maple syrup); distilled alcoholic drinks, baby formula. |  |

**Supplemental Table 2.** **Characteristics of the study participants by quartiles of UPFs (%Kcal) among U.S adults aged 20-59 years.**

|  |  | Quartiles of UPFs (%Kcal) | | | |  |
| --- | --- | --- | --- | --- | --- | --- |
| **Variables** | **Total** | **Q1** | **Q2** | **Q3** | **Q4** | **P value** |
| **Age** | 39.27(0.26) | 40.65(0.39) | 39.55(0.48) | 39.94(0.43) | 37.04(0.37) | < .001 |
| **Sex** |  |  |  |  |  | 0.49 |
| Female | 49.79(0.02) | 51.82(1.35) | 49.20(1.47) | 49.79(1.39) | 48.65(1.57) |  |
| Male | 50.21(0.02) | 48.18(1.35) | 50.80(1.47) | 50.21(1.39) | 51.35(1.57) |  |
| **Race/ethnicity** |  |  |  |  |  | < .001 |
| Non-Hispanic White | 60.01(0.03) | 47.29(2.55) | 63.74(2.02) | 65.14(2.11) | 61.55(2.60) |  |
| Non-Hispanic Black | 11.60(0.01) | 7.18(0.69) | 8.76(0.86) | 12.00(1.18) | 18.01(1.78) |  |
| Mexican American | 10.89(0.01) | 16.84(1.91) | 10.24(1.29) | 8.38(1.06) | 9.15(1.22) |  |
| Others | 17.50(0.01) | 28.70(1.76) | 17.26(1.00) | 14.49(1.04) | 11.30(0.84) |  |
| **Marital status** |  |  |  |  |  | < .001 |
| Never married | 26.03(0.01) | 21.12(1.34) | 23.08(1.45) | 24.89(1.62) | 34.62(1.90) |  |
| Married or living with partner | 60.37(0.03) | 67.55(1.49) | 63.28(1.60) | 60.80(1.88) | 50.66(1.75) |  |
| Widowed, divorced, or separated | 13.59(0.01) | 11.33(0.96) | 13.64(1.04) | 14.31(1.08) | 14.72(0.90) |  |
| **PIR** |  |  |  |  |  | < .001 |
| Low | 23.01(0.01) | 25.45(1.66) | 22.81(1.44) | 22.24(1.33) | 28.25(1.89) |  |
| Middle | 32.09(0.02) | 33.27(1.48) | 33.75(1.55) | 32.45(1.82) | 37.72(1.85) |  |
| High | 38.48(0.02) | 41.28(2.15) | 43.44(1.77) | 45.30(2.42) | 34.02(1.84) |  |
| **Education** |  |  |  |  |  | < .001 |
| College or more | 65.15(0.03) | 63.64(1.79) | 70.56(1.81) | 66.29(1.95) | 59.58(2.01) |  |
| Middle school or lower | 3.70(0.00) | 7.68(1.03) | 3.13(0.37) | 2.33(0.36) | 2.32(0.34) |  |
| High school | 31.14(0.01) | 28.68(1.48) | 26.31(1.67) | 31.38(1.85) | 38.10(2.01) |  |
| **Physical activity** |  |  |  |  |  | 0.01 |
| Active | 59.75(0.02) | 58.13(1.51) | 63.73(1.37) | 58.65(1.40) | 58.14(1.33) |  |
| Inactive | 12.85(0.01) | 12.89(0.81) | 11.74(0.99) | 13.93(1.10) | 12.83(0.82) |  |
| Moderate | 10.74(0.01) | 11.76(1.15) | 11.16(0.84) | 10.00(0.98) | 10.21(0.85) |  |
| Others | 16.66(0.01) | 17.22(1.07) | 13.36(0.90) | 17.43(1.17) | 18.82(1.07) |  |
| **Home status** |  |  |  |  |  | 0.01 |
| Rented | 40.23(0.02) | 40.98(2.02) | 39.95(1.73) | 38.22(1.89) | 44.74(1.81) |  |
| Owned or being bought | 58.15(0.03) | 59.02(2.02) | 60.05(1.73) | 61.78(1.89) | 55.26(1.81) |  |
| **Smoke status** |  |  |  |  |  | < .001 |
| Now | 21.83(0.01) | 17.69(1.16) | 19.52(1.16) | 20.84(1.22) | 28.94(1.15) |  |
| Former | 19.29(0.01) | 19.73(1.27) | 19.81(1.24) | 22.67(1.37) | 14.72(1.02) |  |
| Never | 58.85(0.02) | 62.58(1.65) | 60.67(1.48) | 56.50(1.27) | 56.34(1.27) |  |
| **Drinks** |  |  |  |  |  | < 0.001 |
| Nondrinkers | 22.95(0.01) | 27.06(1.56) | 20.74(1.37) | 22.33(1.25) | 22.41(1.37) |  |
| 1-3 drinks/day | 57.06(0.02) | 53.99(1.77) | 60.08(1.56) | 58.73(1.44) | 54.72(1.63) |  |
| ≥ 4 drinks/day | 19.99(0.01) | 18.95(1.37) | 19.18(1.21) | 18.94(1.15) | 22.87(1.36) |  |
| **Cancer** | 5.097(0.004) | 4.66(0.71) | 5.19(0.68) | 5.58(0.72) | 4.85(0.65) | 0.8 |
| **Stroke** | 1.264(0.001) | 0.92(0.26) | 1.38(0.30) | 1.24(0.24) | 1.46(0.34) | 0.58 |
| **Heart attack** | 1.213(0.001) | 1.13(0.28) | 1.37(0.27) | 1.01(0.27) | 1.34(0.28) | 0.74 |
| **Congestive heart failure** | 0.772(0.001) | 0.52(0.15) | 0.60(0.17) | 0.67(0.18) | 1.29(0.26) | 0.03 |
| **Coronary heart disease** | 0.952(0.002) | 0.91(0.32) | 1.05(0.28) | 0.88(0.21) | 0.96(0.23) | 0.96 |
| **Angina** | 0.99(0.001) | 0.74(0.24) | 1.02(0.30) | 0.90(0.22) | 1.24(0.34) | 0.63 |
| **Hypertension** | 8.857(0.004 | 9.31(0.64) | 8.41(0.74) | 9.50(0.83) | 8.23(0.57) | 0.42 |
| **DM** | 27.26(0.01) | 26.63(1.42) | 26.09(1.49) | 27.81(1.35) | 28.43(1.27) | 0.58 |
| **Energy (kcal)** | 2241.16(11.65) | 2069.28(23.01) | 2202.03(24.30) | 2328.93(32.62) | 2336.09(25.41) | < .001 |
| **Protein(g)** | 86.24(0.63) | 88.59(1.18) | 89.10(1.32) | 87.71(1.37) | 79.59(0.99) | < .001 |
| **eGFR** | 101.57(0.39) | 102.92(0.61) | 100.23(0.59) | 100.55(0.64) | 102.91(0.55) | < 0.001 |
| **BMI** | 28.71(0.13) | 28.15(0.21) | 28.44(0.16) | 28.75(0.22) | 29.42(0.23) | < 0.001 |
| **Weight (Kg)** | 82.01(0.36) | 79.04(0.63) | 81.68(0.52) | 82.59(0.59) | 84.30(0.60) | < .001 |
| **ALM/BMI** | 0.82(0.00) | 0.80(0.01) | 0.82(0.01) | 0.82(0.01) | 0.82(0.01) | < 0.001 |
| **FNIH** | 7.655(0.005) | 8.76(0.78) | 7.07(0.67) | 7.38(0.79) | 7.61(0.90) | 0.41 |
| **EWGSOP2** | 15.69(0.01) | 17.16(1.09) | 15.60(1.02) | 14.80(1.27) | 15.48(1.30) | 0.51 |
| **AWGS** | 8.12(0.01) | 9.22(0.83) | 7.74(0.79) | 6.97(0.75) | 8.81(0.76) | 0.12 |
| **IWGS** | 12.25(0.01) | 14.01(1.06) | 11.30(0.90) | 11.32(1.00) | 12.74(0.92) | 0.12 |

Weighted Mean +/- Se and Student's t-test for continuous variables. Weighted %, mean (95% CI), and Cochran-Mantel-Haenszel Chi-square test for categorical variables.

**Supplemental Table 3.** Multivariable associations between quartiles of UPFs (%Gram) and low muscle mass defined by FNIH.

|  | **Quartiles of UPFs (%Gram)** | | | |  |
| --- | --- | --- | --- | --- | --- |
| **Character** | Q1 | Q2 | Q3 | Q4 | P for trends |
| **ALM/BMI** |  |  |  |  |  |
| **Model 1** | Ref. | 0.017(0.002,0.032) | 0.029(0.014,0.044) | 0.039(0.024,0.054) | <.001 |
| **Model 2** | Ref. | -0.001(-0.011, 0.009) | -0.015(-0.026, -0.004) | -0.027(-0.037, -0.018) | <.001 |
| **Model 3** | Ref. | -0.005(-0.013, 0.004) | -0.016(-0.027, -0.006) | -0.023(-0.032, -0.013) | <.001 |
| **Low muscle mass (FNIH)** |  |  |  |  |  |
| **Model 1** | Ref. | 1.11(0.79,1.55) | 1.17(0.85,1.60) | 1.30(0.99,1.72) | 0.076 |
| **Model 2** | Ref. | 1.18(0.83,1.68) | 1.32(0.96,1.82) | 1.67(1.29,2.17) | <0.001 |
| **Model 3** | Ref. | 1.26(0.85,1.86) | 1.38(0.95,2.02) | 1.82(1.29,2.58) | 0.002 |

Model 1: adjusted for none.

Model 2: adjusted for age and race/ethnicity. ALM/BMI was additionally adjusted for sex.

Model 3: adjusted for age, ethnicity, PIR, marital status, home status, education, physical activity, smoke status, drinks, eGFR, UACR, hypertension, DM, angina, coronary heart disease, congestive heart failure, heart attack, stroke, cancer, energy(kcal), and protein(g). ALM/BMI was additionally adjusted for sex.

*P for trends were calculated by using the median value of each quartile as a continuous variable in each model.

Ref, reference.

**Supplemental Table 4.** Multivariable associations between quartiles of UPFs (%Gram) and low muscle mass defined by EWGSOP2, AWGS2, and IWGS.

|  | **Quartiles of UPFs (%Kcal)** | | | |  |
| --- | --- | --- | --- | --- | --- |
| **Characters** | **Q1** | **Q2** | **Q3** | **Q4** | **P for trends^*^** |
| **Low muscle mass/EWGSOP2** |  |  |  |  |  |
| **Model 1** | Ref. | 1.21(0.90,1.62) | 1.22(0.93,1.59) | 1.33(0.94,1.87) | 0.092 |
| **Model 2** | Ref. | 1.22(0.92,1.62) | 1.20(0.91,1.58) | 1.54(1.09,2.19) | 0.017 |
| **Model 3** | Ref. | 1.34(0.99, 1.83) | 1.15(0.85, 1.56) | 1.50(1.08, 2.08) | 0.041 |
| **Low muscle mass/AWGS2** |  |  |  |  |  |
| **Model 1** | Ref. | 1.00(0.73,1.39) | 0.95(0.71,1.29) | 1.34(1.00,1.79) | 0.076 |
| **Model 2** | Ref. | 0.99(0.72,1.36) | 0.93(0.69,1.24) | 1.42(1.05,1.93) | 0.046 |
| **Model 3** | Ref. | 1.25(0.89,1.76) | 1.07(0.78,1.48) | 1.68(1.18,2.40) | 0.019 |
| **Low muscle mass/IWGS** |  |  |  |  |  |
| **Model 1** | Ref. | 0.94(0.71,1.25) | 1.04(0.79,1.38) | 1.27(0.97,1.67) | 0.059 |
| **Model 2** | Ref. | 0.93(0.70,1.24) | 1.02(0.76,1.36) | 1.38(1.04,1.84) | 0.022 |
| **Model 3** | Ref. | 1.08(0.79,1.46) | 1.12(0.80,1.58) | 1.35(0.97,1.87) | 0.082 |

Model 1: adjusted for weight.

Model 2: adjusted for weight, age, sex, and race/ethnicity.

Model 3: adjusted for weight, age, ethnicity, PIR, marital status, home status, education, physical activity, smoke status, drinks, eGFR, UACR, hypertension, DM, angina, coronary heart disease, congestive heart failure, heart attack, stroke, cancer, energy(kcal), and protein(g).

*P for trends were calculated by using the median value of each quartile as a continuous variable in each model.

Ref, reference.
